# Modulation of blood-brain barrier function by a heteroduplex oligonucleotide *in vivo*

**DOI:** 10.1038/s41598-018-22577-2

**Published:** 2018-03-12

**Authors:** Hiroya Kuwahara, Jindong Song, Takahiro Shimoura, Kie Yoshida-Tanaka, Tadahaya Mizuno, Tatsuki Mochizuki, Satoshi Zeniya, Fuying Li, Kazutaka Nishina, Tetsuya Nagata, Shingo Ito, Hiroyuki Kusuhara, Takanori Yokota

**Affiliations:** 1 Department of Neurology and Neurological Science, Graduate School of Medical and Dental Sciences, Tokyo Medical and Dental University (TMDU), 1-5-45 Yushima, Bunkyo-ku, Tokyo, 113-8519 Japan; 2 Center for Brain Integration Research, Tokyo Medical and Dental University (TMDU), 1-5-45 Yushima, Bunkyo-ku, Tokyo, 113-8519 Japan; 3 Laboratory of Molecular Pharmacokinetics, Graduate School of Pharmaceutical Sciences, The University of Tokyo, 7-3-1 Hongo, Bunkyo-ku, Tokyo, 113-0033 Japan; 4 Department of Pharmaceutical Microbiology, Faculty of Life Sciences, Kumamoto University, 5-1 Oe-honmachi, Chuo-ku, Kumamoto, 862-0973 Japan

## Abstract

The blood-brain barrier (BBB) is increasingly regarded as a dynamic interface that adapts to the needs of the brain, responds to physiological changes, and gets affected by and can even promote diseases. Modulation of BBB function at the molecular level *in vivo* is beneficial for a variety of basic and clinical studies. Here we show that our heteroduplex oligonucleotide (HDO), composed of an antisense oligonucleotide and its complementary RNA, conjugated to α-tocopherol as a delivery ligand, efficiently reduced the expression of *organic anion transporter 3* (*OAT3*) gene in brain microvascular endothelial cells in mice. This proof-of-concept study demonstrates that intravenous administration of chemically synthesized HDO can remarkably silence OAT3 at the mRNA and protein levels. We also demonstrated modulation of the efflux transport function of OAT3 at the BBB *in vivo*. HDO will serve as a novel platform technology to advance the biology and pathophysiology of the BBB *in vivo*, and will also open a new therapeutic field of gene silencing at the BBB for the treatment of various intractable neurological disorders.

## Introduction

The blood-brain barrier (BBB) is no longer regarded only as a substantial barrier for drug delivery to the brain, but also as a dynamic interface that adapts to the needs of the brain and responds to physiological changes; the BBB is affected by and can even promote diseases^1–3^. To expand our understanding of how the BBB functions and interacts with its environment, it is important to establish a platform technology to be able to control gene expression in brain microvascular endothelial cells (BMECs), which are core components of the BBB, *in vivo*. Moreover, the ability to manipulate pathological molecules in BMECs can lead to the development of a new class of molecular targeted therapy for a variety of intractable neurological disorders such as multiple sclerosis, Alzheimer’s disease, and stroke^1,2^.

Oligonucleotide-based gene silencing is a useful strategy, which is being actively developed as both an experimental tool and a therapeutic platform, to modulate biological function at the molecular level *in vivo*^4,5^. Among various types of oligonucleotides, antisense oligonucleotide (ASO) and short-interfering RNA (siRNA) have been widely studied as conventional methods^4,5^. To downregulate gene expression in BMECs, ASO has not been employed so far but siRNA was used; siRNA was hydrodynamically injected^6–8^ or delivered along with extracted endogenous lipoprotein^9^. However, hydrodynamic injection is highly invasive because of the volume overload and high hydrostatic pressure involved, and delivery along with extracted endogenous lipoprotein may have adverse effects caused by blood derivatives. Therefore, an alternative simple and safe strategy is needed; the best one would be conventional systemic administration of a chemically synthesized oligonucleotide without any additional delivery vectors.

We recently developed a new “heteroduplex oligonucleotide” (HDO) approach that achieves highly efficient gene silencing *in vivo*^10^. HDO is composed of an ASO as the parent strand, having a gapmer structure (DNA nucleotides flanked by a few locked nucleic acid (LNA) nucleotides^11^), duplexed with the complementary RNA (cRNA). In the previous study^10^, we proved that intravenously administered α-tocopherol-conjugated HDO (Toc-HDO), in which α-tocopherol (a delivery ligand) is covalently conjugated to the 5′-end of the cRNA, binds to serum lipoproteins in blood circulation, and is distributed along the physiological transport pathway of α-tocopherol^12^. When targeted to the liver, the effect of Toc-HDO was as much as 20 times that of the parent ASO^10^. Given that ASOs are being most actively developed among the oligonucleotide agents^13^, our Toc-HDO has a great potential for gene silencing in various organs and tissues *in vivo*.

Here we report a proof-of-concept study that intravenously injected Toc-HDO can efficiently knock down a target molecule in mouse BMECs, and that this novel platform technology for administering a chemically synthesized oligonucleotide without any additional delivery vectors allows modulation of the BBB function *in vivo*.

## Results

### Screening for effective ASO sequences *in vitro*

We selected mouse *Organic anion transporter 3 (OAT3*), almost exclusively expressed in microvascular endothelial cells within the brain^14^, as the target gene for the following reasons: (1) The lack of *OAT3* expression causes no pathologic phenotype in mice^15^; (2) there is an established experimental approach to quantify the transport function of OAT3 in connection with its expression levels at the BBB^16^.

We first designed 31 ASOs targeting mouse *OAT3* mRNA (NM_031194) to select the most effective sequences to be used as the parent strands of HDO (Supplementary Table S1). The ASOs had a gapmer structure of 8–10 DNA nucleotides flanked by 2 or 3 LNA nucleotides, and all internucleotide linkages were modified by phosphorothioate substitution to increase ASO stability in plasma and binding to plasma proteins and thus, ultimately, tissue bioavailability^17^.

We examined the *in vitro* gene-silencing effect of these ASOs by cotransfecting cultured mouse hepatocellular carcinoma (Hepa1–6) cells with a *Renilla* luciferase-fused *OAT3* expression vector and a firefly luciferase expression vector because there were no cultured cells stably expressing endogenous *OAT3*. Among sequences that effectively inhibited *OAT3* expression, we selected sequences No. 1 (93% inhibition), 18 (79% inhibition), and 30 (81% inhibition) (Supplementary Fig. S1).

### Distribution of intravenously injected Toc-HDO in mouse brain

**Figure 1:**
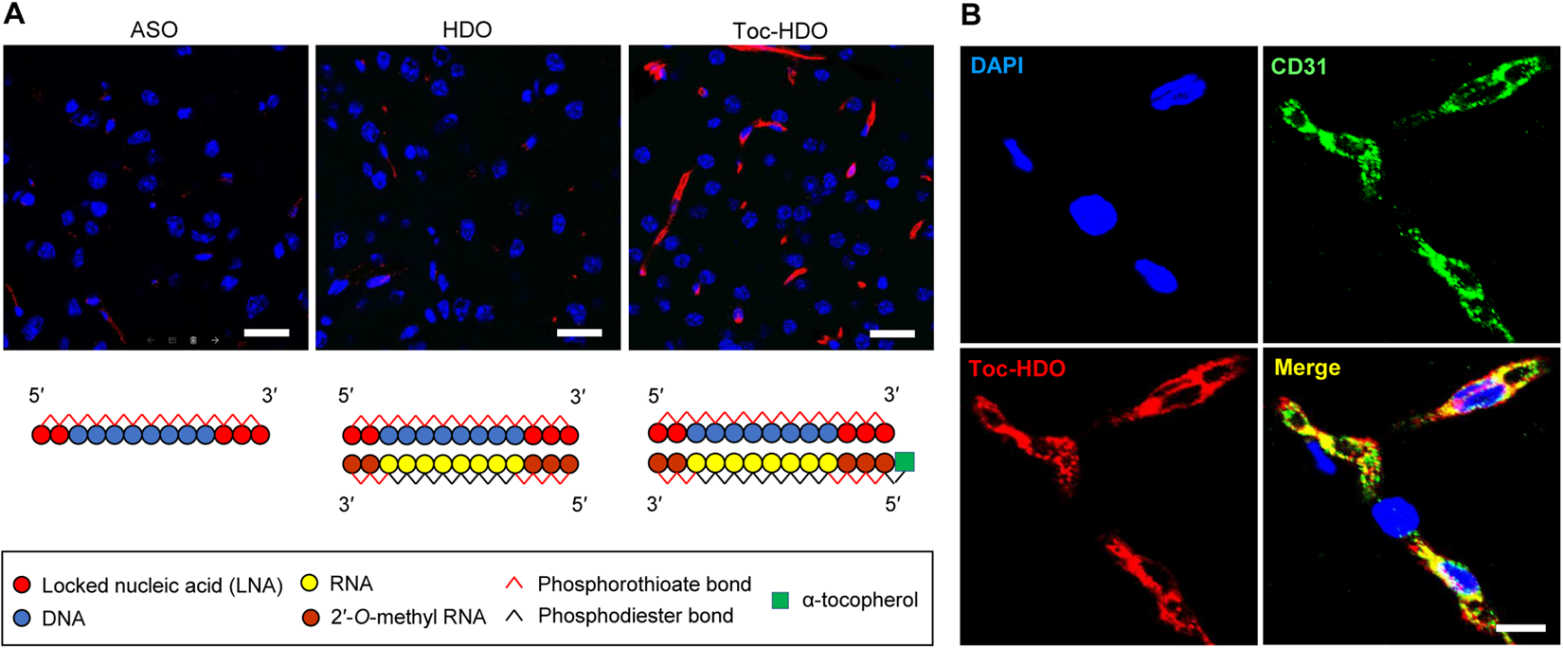
Distribution of intravenously administered oligonucleotides in mouse brain. Confocal laser scanning images of cerebral sections prepared 1 h after an intravenous injection of **(A)** Alexa Fluor 568–labeled ASO, HDO, or **(A,B)** Toc-HDO at doses corresponding to 16 mg/kg of ASO. Sections were stained with DAPI and were immunolabeled with antibody against CD31 (**B** only) (*N* = 3). Red, Alexa Fluor 568. Scale bars: **(A)** 25 µm; **(B)** 10 µm. Schematic illustrations of the construction of ASO, HDO and Toc-HDO are given at the bottom part of **(A)**.

To prepare HDOs, we designed 13- to 16-mer parent ASOs on the basis of sequences No. 1, 18, and 30, and the cRNA complementary to each ASO sequence (Supplementary Table S2). In the cRNA strand, phosphorothioate-modified 2′-*O*-methyl sugar modifications were introduced into the nucleotides complementary to LNA in the ASO strand for protection from exonucleases (Fig. 1A, Supplementary Table S2). We also prepared Toc-HDOs in which α-tocopherol was covalently bound to the 5′-end of the cRNA strand (Fig. 1A).

To examine the distribution of ASO, HDO, and Toc-HDO, we injected these oligonucleotides, labeled with Alexa Fluor 568 at the 5′-end of each ASO strand, into the tail vein at a dose corresponding to 16 mg/kg of ASO, and examined their presence in the brain 1 h after the injection. Injection of ASO or HDO resulted in weak signals (Fig. 1A), whereas injection of Toc-HDO gave robust signals in linear structures, suggesting that Toc-HDO accumulated along the brain microvasculature (Fig. 1A). Fluorescent signals colocalized with BMECs, which were visualized with CD31 antibody (Fig. 1B). Similar signal distribution was seen in all parts of the brain, and almost no fluorescent signals were detected in other brain cell types, such as neurons or glia (Supplementary Fig. S2), indicating that intravenously-administered Toc-HDO could hardly pass through the BBB. Moreover, these signal distributions did not alter when we used the ASO, HDO, and Toc-HDO having a scramble nucleotide sequence that do not target a protein expressed at the BBB, suggesting that the conjugation of α-tocopherol, not the nucleotide sequence, substantially determines the distribution of the oligonucleotides in the brain (Supplementary Fig. S3).

### Gene silencing effect of Toc-HDO in BMECs

**Figure 2:**
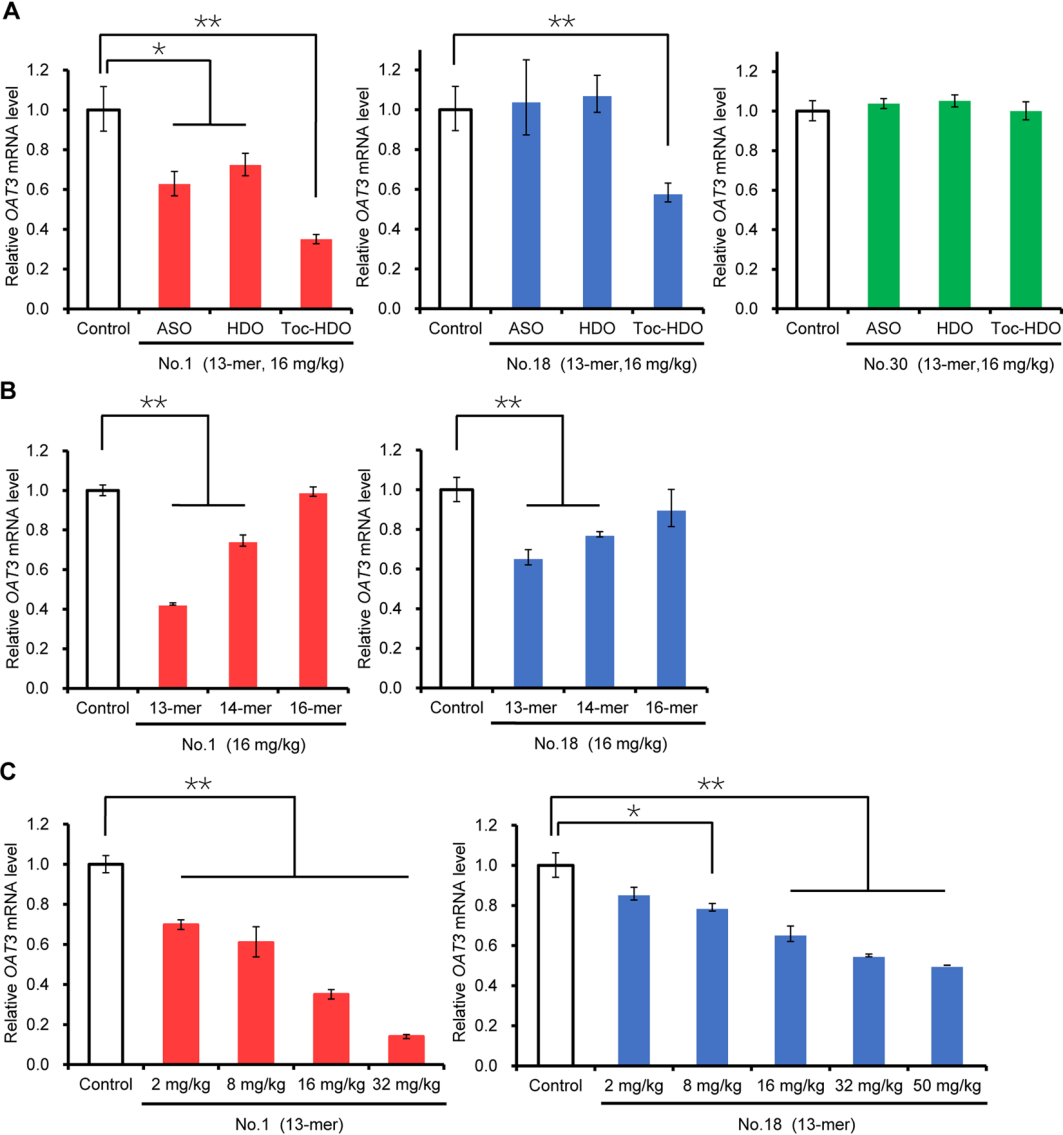
Gene silencing by intravenous administration of *OAT3*-targeting oligonucleotides. *OAT3* mRNA levels measured by quantitative RT-PCR in brain homogenates 72 h after an intravenous injection of **(A)** 13-mer ASO, HDO, or Toc-HDO (No. 1, 18, or 30) at doses corresponding to 16 mg/kg of ASO; **(B)** 13-, 14-, or 16-mer Toc-HDO (No. 1 or 18) at doses corresponding to 16 mg/kg of ASO; **(C)** 13-mer Toc-HDO (No. 1 or 18) at different ASO doses. Data shown are relative to *Claudin-5* mRNA levels and are expressed as mean values ± s.e.m. (*N* = 3, *P < 0.05, **P < 0.01). *P* values were calculated using Student’s two-tailed *t*-test.

First, mice were injected with 13-mer ASO, HDO, or Toc-HDO (No. 1, 18, or 30) at doses corresponding to 16 mg/kg of ASO, and were euthanized 72 h after the injection, and quantitative reverse-transcription PCR (qRT-PCR) was used to determine the *OAT3* mRNA levels in brain homogenates. Toc-HDO (No. 1) was more effective in reducing *OAT3* expression (by 65%) than ASO or HDO with the same sequence (Fig. 2A). Toc-HDO (No. 18) significantly reduced *OAT3* expression (by 42%), but there was no reduction by ASO or HDO (Fig. 2A). Toc-HDO (No. 30), as well as the corresponding ASO and HDO, had no significant effect (Fig. 2A). Based on these results, we decided to test Toc-HDOs (No. 1) and (No. 18) in the following experiments.

Next, we examined how the length of these Toc-HDOs (administered at the same doses) affected the reduction in *OAT3* mRNA levels. The highest reduction was observed with the 13-mer Toc-HDOs (both No. 1 and 18), and the 14-mer Toc-HDOs also showed reduction, whereas the effects of 16-mer Toc-HDOs were not evident (Fig. 2B). These results are in line with our previous data on the effects of the 13-, 14-, and 16-mer Toc-HDO in the liver^10^.

Then we tested the effect of various doses of 13-mer Toc-HDOs. Toc-HDO (No. 1) was most efficient (inhibition by 86% at 32 mg/kg), and its 50% effective dose (ED_50_) was 10.7 mg/kg (Fig. 2C). Toc-HDO (No. 18) demonstrated maximal inhibition (by 50%) at 50 mg/kg (Fig. 2C). These data indicate that intravenously administered 13-mer Toc-HDOs (No. 1 and 18) can downregulate *OAT3* mRNA in BMECs *in vivo*.

**Figure 3:**
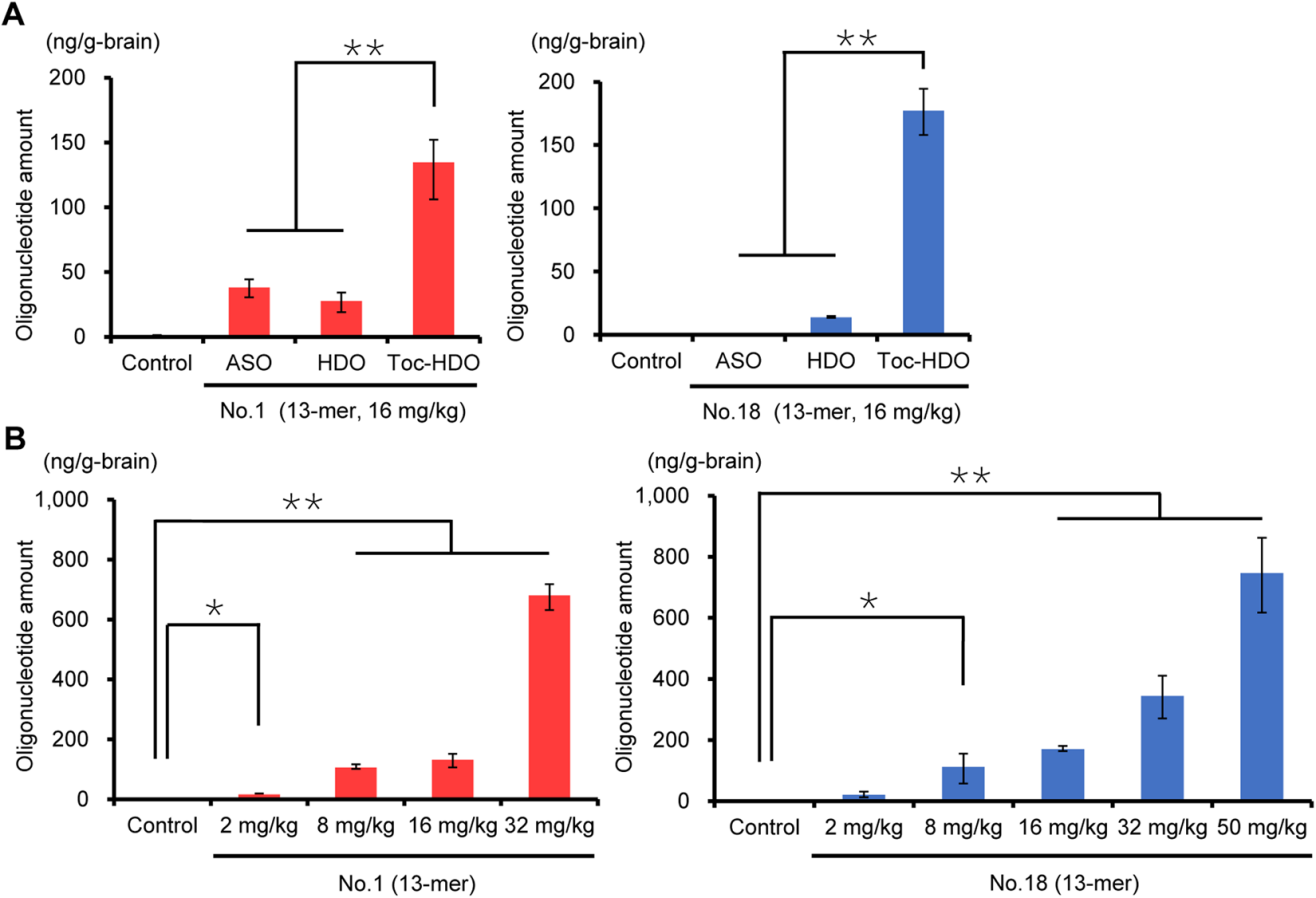
Quantification of the parent ASO strand after intravenous administration of *OAT3*-targeting oligonucleotides. Amounts of the parent ASO strand in the brain measured by quantitative RT-PCR 72 h after an intravenous injection of **(A)** 13-mer ASO, HDO, or Toc-HDO (No. 1 or 18) at doses corresponding to 16 mg/kg of ASO; **(B)** 13-mer Toc-HDO (No. 1 or 18) at different ASO doses. Data shown are relative to mouse *U6* RNA levels and are expressed as mean values ± s.e.m. (*N* = 3, *P < 0.05, **P < 0.01). *P* values were calculated using Student’s two-tailed *t*-test.

To confirm the delivery of intravenously administered oligonucleotides into BMECs, we performed qRT-PCR to quantify the parent ASO strand in the same RNA samples. In mice injected with 13-mer ASO, HDO, or Toc-HDO (16 mg/kg), accumulation of Toc-HDOs (both No. 1 and 18) in the brain was remarkably higher than that of ASOs and HDOs (Fig. 3A). These results are consistent with accumulation of fluorescently labeled oligonucleotides in BMECs (Fig. 1A). Dose-dependent accumulation of 13-mer Toc-HDOs (both No. 1 and 18) was also confirmed (Fig. 3B).

### Adverse effects of Toc-HDO

**Figure 4:**
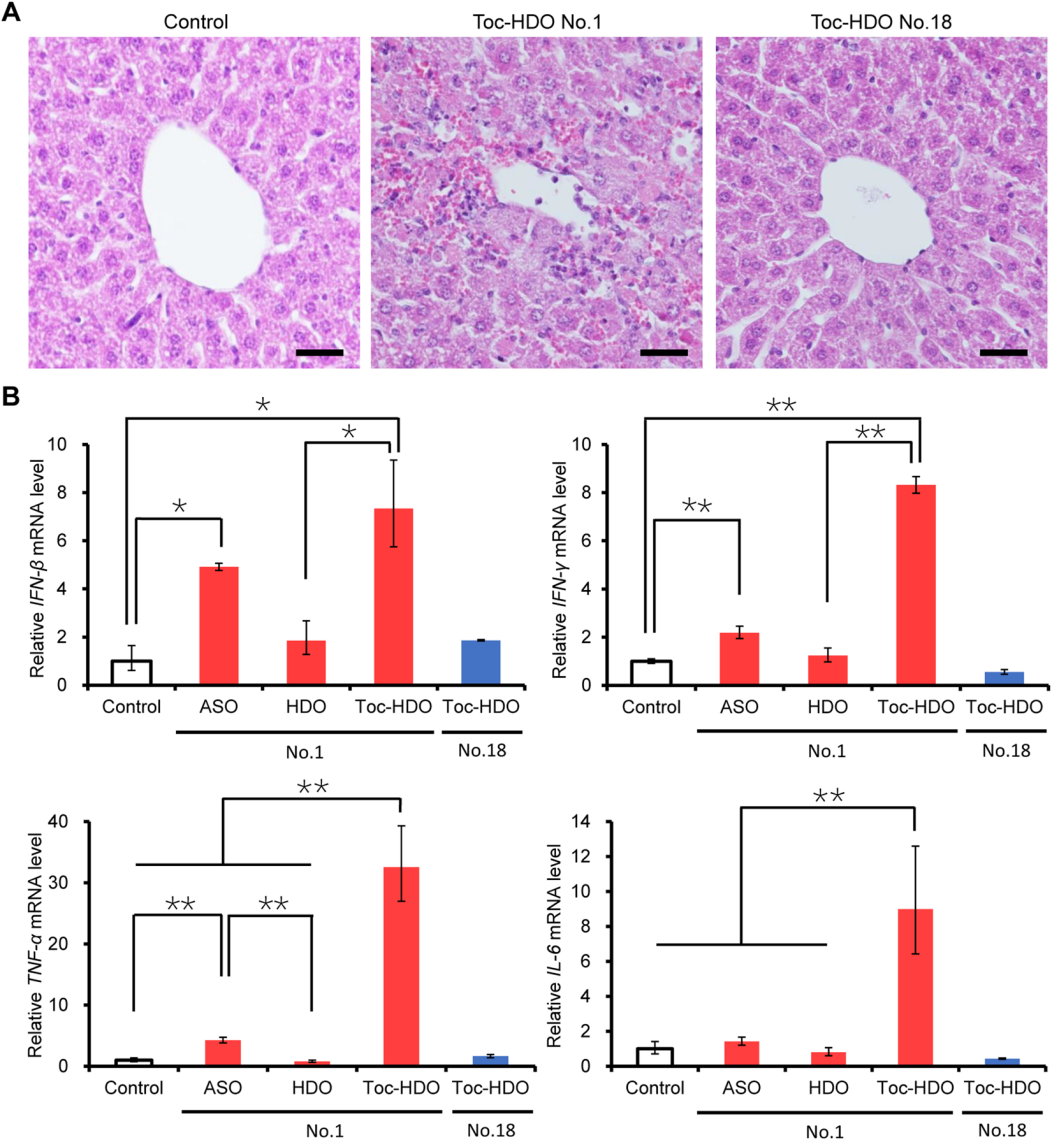
Adverse effects in liver after intravenous administration of Toc-HDO. (**A**) Hematoxylin and eosin staining of liver sections prepared 72 h after an intravenous injection of 13-mer Toc-HDO (No. 1 or 18) at a dose corresponding to 16 mg/kg of ASO. Scale bars = 20 µm. **(B)** *Interferon-β* (*IFN-β*), *Interferon-γ* (*IFN-γ*), *Tumor necrosis factor-α* (*TNF-α*), and *Interleukin-6* (*IL-6*) mRNA levels measured by quantitative RT-PCR in liver homogenates 72 h after an intravenous injection of 13-mer ASO, HDO, or Toc-HDO (No. 1), or 13-mer Toc-HDO (No. 18). All injection doses corresponded to 16 mg/kg of ASO. Data shown are relative to *Glyceraldehyde-3-phosphate dehydrogenase* (*Gapdh*) mRNA levels and are expressed as mean values ± s.e.m. (*N* = 3, *P < 0.05, **P < 0.01). *P* values were calculated using Student’s two-tailed *t*-test.

We evaluated the potential adverse effects of Toc-HDOs to check if we could proceed with the assessment of OAT3 function at the BBB. We noticed by a visual observation that mice injected with Toc-HDO (No. 1) at 16 or 32 mg/kg moved slowly, whereas all mice injected with Toc-HDO (No. 18) remained active even after the injection at doses higher than 16 mg/kg. Serum chemistry analyses after Toc-HDO (No. 1) injection showed severe liver dysfunction (as evidenced by elevated levels of total bilirubin, aspartate aminotransferase, alanine aminotransferase, and alkaline phosphatase), which worsened with the increase in the injected dose, and mild renal dysfunction (Supplementary Table S3). Hematoxylin and eosin staining of liver tissues revealed lymphocyte infiltration with focal bleeding in mice injected with Toc-HDO (No. 1) (Fig. 4A), whereas mice injected with Toc-HDO (No. 18) did not show overt abnormalities in serum chemistry or histological analyses at any doses tested (Supplementary Table S3, Fig. 4A).

Then we examined the expression of pro-inflammatory cytokines in mouse liver. We found the induction of *Interferon-β* (by 7.3-fold), *Interferon-γ* (by 8.3-fold), *Tumor necrosis factor-α* (by 33-fold), and *Interleukin-6* (by 9.0-fold) after Toc-HDO (No. 1) injection in comparison with the control mice, whereas Toc-HDO (No. 18) had no effect (Fig. 4B). The liver toxicity of Toc-HDO (No. 1) might be associated mainly with the immunostimulatory effect of the parent ASO itself because the parent ASO alone also induced most of these cytokines (Fig. 4B). Although hematoxylin and eosin staining of the brain tissues revealed no infiltration of inflammatory cells in mice injected with Toc-HDO (No. 1) at 16 mg/kg (Supplementary Fig. 4), we concerned the possibility of Toc-HDO (No. 1) causing some secondary effects on the brain and therefore decided to use only Toc-HDO (No. 18) in the following experiments.

### Modulation of target protein function at the BBB by Toc-HDO

**Figure 5:**
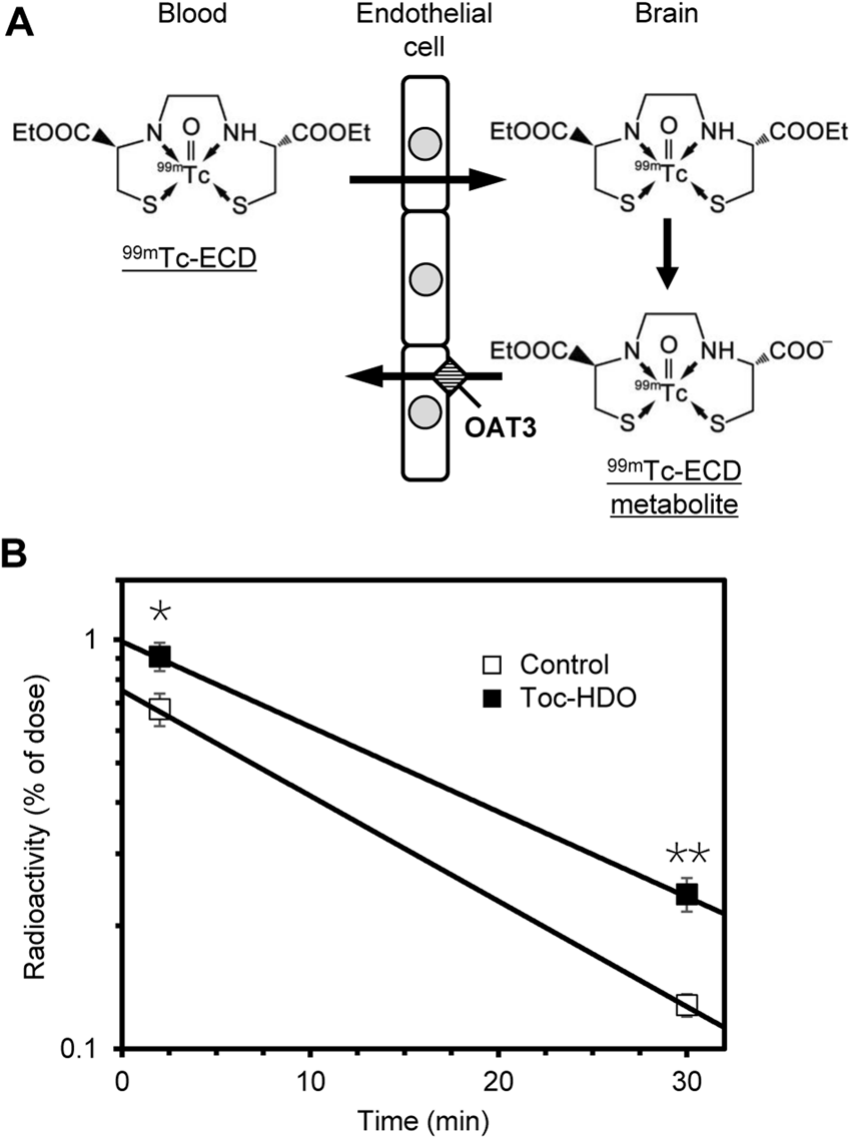
Retention of an OAT3 substrate in the brain after intravenous administration of Toc-HDO. (**A**) Schematic representation of the transport of ^99m^Tc-ECD and its metabolite in the brain. **(B**) Radioactivity of the ^99m^Tc-ECD metabolite in the brain measured 2 or 30 min after an intravenous injection of ^99m^Tc-ECD into mice that were injected 72 h earlier with PBS (open squares) or 13-mer Toc-HDO (No. 18) at a dose corresponding to 50 mg/kg of ASO (closed squares). Data are expressed as mean values ± s.e.m. (*N* = 5, *P < 0.05, **P < 0.01); regression lines show the retention of the ^99m^Tc-ECD metabolite in the brain. *P* values were calculated using Student’s two-tailed *t*-test.

We investigated the effect of Toc-HDO (No. 18) at the protein level. To analyze the efflux transport function of OAT3 at the BBB, we used a technetium-99m complex with *N*,*N′*-1,2-ethylenediylbis-L-cysteine diethyl ester (^99m^Tc-ECD), a metabolite of which is transported by OAT3 from brain to blood at the abluminal plasma membrane of BMECs^16^ (Fig. 5A).

We intravenously injected 13-mer Toc-HDO (No. 18) at 50 mg/kg since this dose was expected to be more effective than the other lower doses tested (Fig. 2C). After 72 h, we intravenously injected ^99m^Tc-ECD and collected the brain after a further 2 or 30 min. The radioactivity of the ^99m^Tc-ECD metabolite in the brain was significantly higher in mice injected with Toc-HDO than in the control mice at both 2 and 30 min (Fig. 5B). The OAT3-mediated efflux rate was lower by 24% in mice injected with Toc-HDO than in the control mice. These results indicate that intravenously administered Toc-HDO reduces the efflux transport function of OAT3 at the BBB, leading to the retention of the OAT3 substrate in the brain.

### Cumulative gene silencing effect by repeated injections of Toc-HDO

**Figure 6:**
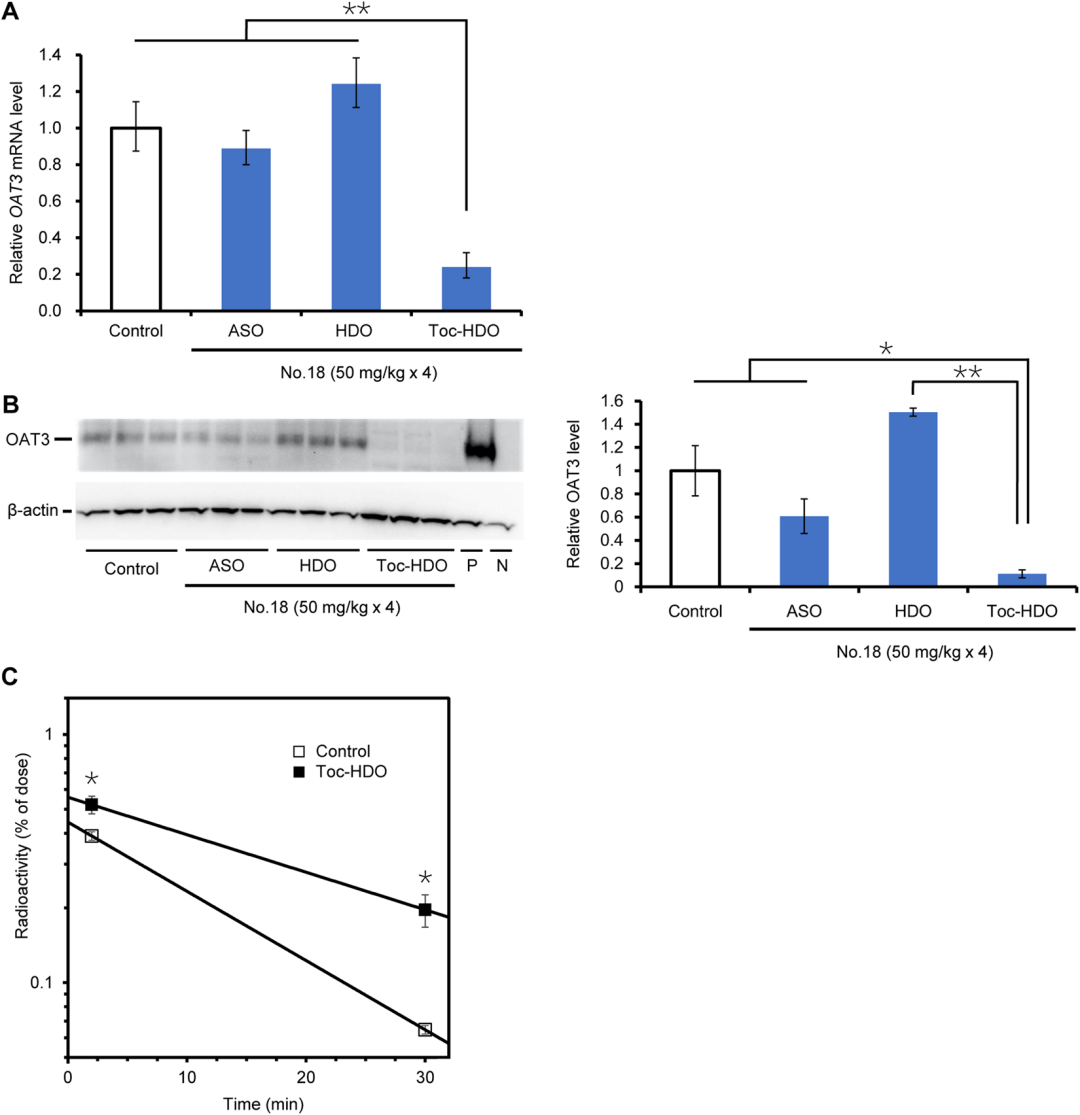
Cumulative gene silencing effect by repeated administration of Toc-HDO. (**A**) *OAT3* mRNA levels measured by quantitative RT-PCR in brain homogenates after four intravenous injections (at 1-week intervals) of 13-mer ASO, HDO, or Toc-HDO (No. 18) at doses corresponding to 50 mg/kg of ASO. Brains were obtained 72 h after the last injection. Data shown are relative to *CD31* mRNA levels and are expressed as mean values ± s.e.m. (*N* = 3, **P < 0.01). *P* values were calculated using Student’s two-tailed *t*-test. **(B)** Western blot analysis of the OAT3 protein in the microvascular fraction of the brains of mice treated as in **(A)**. β-actin was used as an internal control. P, positive control (kidney sample from a wild-type mouse); N, negative control (kidney sample from an *OAT3*-knockout mouse). Quantification of the OAT3 band densities are expressed as mean values ± s.e.m. (*N* = 3, *P < 0.05, **P < 0.01). *P* values were calculated using Student’s two-tailed *t*-test. **(C)** Radioactivity of the ^99m^Tc-ECD metabolite in the brain measured 2 or 30 min after an intravenous injection of ^99m^Tc-ECD into mice that were injected four times at 1-week intervals, by up to 72 h before, with PBS (open squares) or 13-mer Toc-HDO (No. 18) at a dose corresponding to 50 mg/kg of ASO (closed squares). Data are expressed as mean values ± s.e.m. (*N* = 5, *P < 0.05); regression lines show the retention of the ^99m^Tc-ECD metabolite in the brain. *P* values were calculated using Student’s two-tailed *t*-test.

To examine whether we could increase the gene silencing effect by repeating Toc-HDO administration, we intravenously injected 13-mer ASO, HDO, or Toc-HDO (No. 18) at 50 mg/kg four times at 1-week intervals, and analyzed *OAT3* mRNA levels 72 h after the last injection. *OAT3* mRNA levels were remarkably decreased (by 76%) by repeated injections of Toc-HDO (Fig. 6A), which were more efficient than a single injection (50% decrease; Fig. 2C). The *OAT3* mRNA levels were not decreased by ASO or HDO, suggesting that the cumulative gene silencing effect was achieved only with Toc-HDO.

We were unable to detect the OAT3 protein in BMECs by western blot analyses of whole-brain homogenates, likely because the number of BMECs is small in the entire brain. Therefore, we obtained a brain fraction containing enriched microvessels using a dextran gradient technique^9^ and analyzed this fraction. The OAT3 protein level was remarkably reduced after the injections of Toc-HDO (No. 18) (by 89%) but not ASO or HDO (Fig. 6B, Supplementary Fig. S5). These data indicate that intravenous administration of Toc-HDO is highly efficient and results in a knockdown of the OAT3 protein in BMECs after repeated injections.

Furthermore, we also evaluated the effect of repeating Toc-HDO administration on the efflux transport function of OAT3 at the BBB. Specifically, we intravenously injected 13-mer Toc-HDO (No. 18) at 50 mg/kg four times at 1-week intervals, and 72 h after the last injection, we intravenously injected ^99m^Tc-ECD and collected the brain after a further 2 or 30 min. As with the previous experiment of a single injection, the radioactivity of the ^99m^Tc-ECD metabolite in the brain was higher in mice injected with Toc-HDO than in the control mice at both 2 and 30 min (Fig. 6C). Most notably, the OAT3-mediated efflux rate was much lower by 55% in mice repeatedly injected with Toc-HDO than in the control mice (Fig. 6C), which demonstrated a cumulative effect of Toc-HDO on the OAT3 activity.

To examine whether repeated injections of Toc-HDO adversely influence the BBB, we performed the following two experiments. The first one is a well-known screening experiment using Evans blue to investigate whether the BBB was disrupted^18^. We intravenously injected 13-mer Toc-HDO (No. 18) at 50 mg/kg four times at 1-week intervals, and 72 h after the last injection, we intravenously injected 2% Evans blue solution. A visual observation of the brain after a further 24 h revealed no massive extravasation of Evans blue into the brain (Supplementary Fig. 6), suggesting no overt disruption of the BBB. Another experiment is an evaluation of the brain histology after repeated injections of Toc-HDO. Hematoxylin and eosin staining of the brain tissues obtained 72 h after the last injection of Toc-HDO (No. 18) revealed no abnormalities (Supplementary Fig. 7), suggesting no apparent adverse effects on the BBB microscopically, either.

## Discussion

In the current study, we demonstrated that the 13-mer Toc-HDO (No. 18) targeting *OAT3* mRNA was efficiently delivered into BMECs upon intravenous administration to mice to induce significant suppression of OAT3 function. Its suppressive effect was cumulative, suggesting the possibility of further optimization of dosing schedule to potentiate the effect. Given that this is a proof-of-concept study, we consider Toc-HDO a reasonable platform technology for modulating BBB function *in vivo*.

The techniques to modulate BBB function at the molecular level *in vivo* would be beneficial for a variety of basic and clinical studies, in particular to advance our understanding of the biology and pathophysiology of the BBB^1^. Blocking antibodies delivered systemically can be used to inhibit some specific proteins expressed in BMECs, but they mainly target proteins located on the surface of luminal plasma membranes. In contrast, oligonucleotide agents can also inhibit the production of proteins located on the abluminal plasma membranes, such as receptors and transporters, and intracellular molecules including non-coding RNAs involved in large network of biological and pathological processes^5,19^. Therefore, Toc-HDO can be an invaluable technology to foster a broad range of basic studies relevant to the BBB.

BBB modulation may provide a potential platform for drug development to treat many intractable neurological disorders, because BMECs express various disease-specific pathological molecules^1,2^. In multiple sclerosis, for example, a variety of cell adhesion and signaling molecules are involved in lymphocyte migration from the blood into the brain across the BBB, which is the initial step of pathogenesis^20,21^. The most pivotal process in this step is the adhesion of very late antigen-4 (the synonym of α4β1-integrin) on lymphocytes to vascular cell adhesion molecule-1 (VCAM-1) on BMECs^22^. Natalizumab, a monoclonal antibody against α4-integrin, blocks the entrance of pathogenic lymphocytes into the brain and improves the outcome by preventing relapses in a substantial number of multiple sclerosis patients^23–25^. Therefore, the pathological molecules of BMECs, such as VCAM-1, can be targeted to treat multiple sclerosis.

In Alzheimer’s disease, the receptor for advanced glycation end products (RAGE) expressed in BMECs mediates the transport of the neurotoxic amyloid-β peptide from the blood into the brain, leading to oxidative stress and neuroinflammation^26^. Expression of RAGE in BMECs of Alzheimer’s disease patients is approximately 2.5 times that in age-matched control groups^27^. Inhibition of RAGE in BMECs is expected to alleviate disease pathology^28^, suggesting RAGE as a target to treat Alzheimer’s disease. Tumor necrosis factor-related apoptosis-inducing ligand (TRAIL) death receptors on BMECs might also be targeted because oligomeric amyloid-β peptides specifically interact with these receptors to induce extrinsic apoptotic pathways, whereas silencing of the receptors by RNA interference protects BMECs from apoptosis^29^.

Therapeutic strategy based on BBB modulation might become applicable to many other disorders such as stroke; epilepsy; traumatic brain injury; and neuroinfectious, neuroinflammatory, and neurodegenerative diseases^1,2^ when the pathomechanisms of these disorders in BMECs are elucidated in more detail. The efflux transporters in BMECs, such as P-glycoprotein, breast cancer resistance protein, and multidrug resistance-associated protein-4 might also be targeted, because many low-molecular-weight drugs now clinically available are substrates of these transporters and are therefore promptly eliminated into the blood even if these drugs can reach the brain across the BBB^30^. Inhibition of these efflux transporters is expected to increase the content of the drugs in the brain and contribute to their higher efficacy.

To enable the general use of our platform technology (Toc-HDO), the delivery mechanism of Toc-HDO into BMECs should be discussed. Using fluorescence correlation spectroscopy and fast protein liquid chromatography, we previously found that intravenously administered Toc-HDO binds to lipoproteins in mouse serum, particularly to high-density lipoprotein (HDL), and is distributed along the physiological transport pathway of α-tocopherol^10^. ASO and HDO alone, in the absence of a lipid ligand of α-tocopherol, are rather hydrophilic and therefore, these oligonucleotides can hardly bind to lipoproteins and poorly interact with cellular membranes comprised of lipid bilayer, resulting in the limited uptake into BMECs. The uptake of Toc-HDO into hepatocytes is mediated by low-density lipoprotein receptor (LDLR)^10^, suggesting that a similar mechanism of uptake via lipoprotein receptors could occur in other organs and tissues. Given that BMECs express lipoproteins such as LDLR, scavenger receptor class B type 1 (SR-B1), and LDLR-related proteins^31,32^, and that the uptake of α-tocopherol into the brain is mediated by SR-B1^31^, the delivery of Toc-HDO bound to HDL into BMECs might be speculated to occur at least in part via lipoprotein receptors. Because most lipoproteins in the brain are considered to originate from the excretion by glial cells, with a minimal supply from the blood circulation across the BBB^33,34^, Toc-HDO would dissociate from HDL after the uptake by BMECs and would be released from endosomes into the BMEC cytosol.

We previously showed that direct conjugation of α-tocopherol to ASO abolishes intracellular ASO activity, probably because α-tocopherol interferes with the access of the ASO to the target mRNA in the nucleus^10,35^. We also showed that cRNA of Toc-HDO is cleaved by intracellular endonucleases and is released from the parent ASO, which is a key processing step for ASO activity^10^. Assuming that the mechanism and efficiency of the intracellular processing of HDO do not depend on the delivery ligands bound to cRNA, some ligands might improve delivery to BMECs. The destination of intravenously administered Toc-HDO is mostly liver; our previous study showed an ED_50_ of 0.038 mg/kg in the liver upon a single injection of Toc-HDO^10^, whereas the current study indicated that the ED_50_ of Toc-HDO exceeded 10 mg/kg in BMECs. Decreasing the injected dose of oligonucleotide would reduce the possible adverse effects and thus enhance the practical applicability of our HDO technology *in vivo*.

To decrease the injected dose of oligonucleotide, much more effort should be devoted to the search for nucleotide sequences that are extremely effective and safe. In the current study, we screened 31 ASO sequences *in vitro* and selected 3 effective sequences for the experiments *in vivo*, leading to an identification of only 1 sequence that was demonstrated to be effective without noticeable adverse effects *in vivo*. If we could screen hundreds or thousands of nucleotide sequences at the initial phase of study, we might have identified better sequences and decreased the injected dose. Moreover, conjugating a ligand to enhance the tropism towards BMEC would also help to decrease the dose of oligonucleotide, just like N-acetylgalactosamine ligand for the targeted delivery to liver via hepatocyte-specific asialoglycoprotein receptor^36,37^. Among the various candidate ligands previously studied for enhancing the tropism towards BMEC, glucose, the main energy source in the brain, is notable because glucose transporter-1 is expressed at a remarkably high level in BMECs than in many other tissues. Actually, we recently constructed a highly preferential and efficient drug delivery platform into and even crossing the BMECs by targeting the glucose transporter-1^38^. Another possible strategy is utilizing polypeptide ligands selected from a phage library by *in vivo* panning; the delivery of a gene to BMEC was increased by inserting the polypeptide into the binding site of the adeno-associated viral vector to its receptor^39^.

Liver dysfunction is an important concern for the development of all antisense therapeutic modalities^40^. First, sequence-dependent and hybridization-dependent off-target effects^41^ can be observed with our HDO. Our previous microarray gene expression analysis showed several down-regulated genes with sequences similar to that of the target gene; this effect was predicted to be caused by off-target hybridization of HDO^10^. Second, sequence-dependent and hybridization-independent immunostimulatory adverse effects mediated by activation of Toll-like receptors^42,43^ can be induced by HDO. In the current study, ASO (No. 1) stimulated the expression of pro-inflammatory cytokines in the liver (Fig. 4B). Finally, sequence-independent and hybridization-independent chemical properties of nucleotide analogs and chemical modifications can cause liver dysfunction^44^. However, Toc-HDO (No. 18), which had the similar chemical properties as Toc-HDO (No. 1), showed no abnormalities in the liver, suggesting that chemical toxicity may not contribute to liver dysfunction.

In summary, we efficiently silenced a target molecule expressed in BMECs by intravenous administration of Toc-HDO. Knockdown of the target was verified at the mRNA and protein levels, and modulation of target protein function at the BBB was demonstrated. We believe that our novel platform technology based on HDO will advance a variety of basic and clinical studies on biology and pathophysiology of the BBB, and will also open a new therapeutic field of gene silencing at the BBB for the treatment of many intractable neurological disorders.

## Methods

### Design and synthesis of oligonucleotides

A series of 13- to 14-mer ASOs designed to target mouse *OAT3* mRNA (NM_031194) and an unrelated ASO are shown in Supplementary Table S1. The ASOs had the gapmer structure of 8–10 DNA nucleotides flanked by 2 or 3 LNA nucleotides, and all internucleotide linkages were modified by phosphorothioate substitution. For immunohistochemical examinations, Alexa Fluor 568 was covalently bound to the 5′-ends of the ASOs. All ASOs were synthesized by Gene Design (Osaka, Japan).

A series of 13-, 14-, and 16-mer cRNAs designed to be complementary to the ASO sequences are shown in Supplementary Table S2. Phosphorothioate-modified 2′-*O*-methyl sugar modifications were introduced into the RNAs complementary to LNA in the ASO strand. To produce Toc-HDO, α-tocopherol was covalently bound to the 5′-end of the cRNA strand. All cRNAs were synthesized by Gene Design or Hokkaido System Science (Sapporo, Japan).

To generate HDO and Toc-HDO, equimolar amounts of ASO and cRNA strands in PBS were heated at 95 °C for 5 min and slowly cooled to room temperature.

### Mouse studies

Wild-type C57BL/6 mice aged 7–10 weeks (Oriental Yeast, Tokyo, Japan) were kept on a 12-h light/dark cycle in a pathogen-free animal facility with free access to food and water. ASO, HDO, or Toc-HDO was administered to the mice by tail vein injection; doses were chosen in accordance with their body weight. All oligonucleotides were formulated in PBS, which was also used as the control. The oligonucleotides were administered by a single injection or repeated injections (four times at 1-week intervals). For postmortem analyses, mice were deeply anesthetized with intraperitoneally administered pentobarbital (60 mg/kg) and then killed by transcardiac perfusion with PBS after confirming the absence of the blink reflex.

Animal experiments were performed at Tokyo Medical and Dental University, except for the functional assay of OAT3 in the brain, which was performed at The University of Tokyo. At least three mice per experiment were used. All experimental protocols were approved by the Institutional Animal Care and Use Committee of Tokyo Medical and Dental University (No. 0170179A) and The University of Tokyo (No. 55). The procedures were in accordance with the ethical and safety guidelines for animal experiments of Tokyo Medical and Dental University and The University of Tokyo.

### *In vitro* studies

Mouse *OAT3* cDNA was subcloned from pGEM-HEN/Roct (OAT3) into the *Renilla* luciferase expression vector psiCHECK-1 (Promega, Fitchburg, WI). Hepa1–6 cells were transfected with 20 ng of the *Renilla* luciferase-fused OAT3 expression vector, 20 ng of the firefly luciferase expression vector pGL3 (Promega), and ASO (10 nmol/l) per well of 24-well plates with Lipofectamine 2000 (Thermo Fisher Scientific, Waltham, MA). Luciferase activities were analyzed 24 h after transfection by using the Dual Luciferase System (Promega). *Renilla* luciferase activity was normalized to firefly luciferase activity.

### Immunohistochemical analyses

Alexa Fluor 568–labeled ASO, HDO, or Toc-HDO was injected (at doses corresponding to 16 mg/kg of ASO) into the tail veins of mice. After 1 h, brains were fixed in 4% paraformaldehyde/PBS for 12 h, snap-frozen in liquid nitrogen and sectioned (14 µm) with a LEICA CM3050 S cryostat (Leica Microsystems, Wetzlar, Germany). The sections were stained with DAPI (4′,6-diamidino-2-phenylindole) to visualize the nuclei and were immunolabeled with antibody against CD31 (1:200, sc-18916, Santa Cruz Biotechnology, Santa Cruz, CA), Tuj1 (1:100, 801201; BioLegend, San Diego, CA), Iba1 (1:300, 019-19741; Wako Pure Chemical Industries, Osaka, Japan), and glial fibrillary acidic protein (1:200, ab16997; Abcam, Cambridge, UK) to visualize vascular endothelial cells, neurons, microglia, and astrocytes, respectively. This was followed by incubation with an Alexa Fluor 488–conjugated secondary antibody (1:100, A11001, A11006, or A11008, Thermo Fisher Scientific). All images were acquired with an A1R confocal laser scanning microscope (Nikon, Tokyo, Japan).

### Quantitative reverse transcription–PCR

Total RNA was extracted from mouse brain by using Isogen (Nippon Gene, Tokyo, Japan). To detect mRNA, RNA (1.5 μg) was reverse transcribed with Transcriptor Universal cDNA Master (Roche Diagnostics, Mannheim, Germany). To detect short oligonucleotides, including the parent ASO strand, qRT-PCR analysis was performed by using the TaqMan MicroRNA Reverse Transcription Kit (Thermo Fisher Scientific) and a Light Cycler 480 Real-Time PCR Instrument (Roche Diagnostics). The primers and probes for mouse *OAT3* (Mm00459534_m1), *Claudin-5* (Mm00727012_s1), *CD31 (*Mm01242584_m1*)*, *U6* (001973), *Interferon-β* (*IFN-β*; Mm00439546_s1), *Interferon-γ* (*IFN-γ*; Mm00801778_m1)*, Tumor necrosis factor-α* (*TNF-α*; Mm00443258_m1), *Interleukin-6* (*IL-6*; Mm00446190_m1), and *Glyceraldehyde-3-phosphate dehydrogenase (Gapdh*; 4352932E), and each ASO strand were designed by Thermo Fisher Scientific. Relative *OAT3* mRNA levels were calculated in comparison with *Claudin-5* or *CD31* mRNA levels, which were used as BMEC-specific internal controls. Relative *IFN-β*, *IFN-γ*, *TNF-α*, and *IL-6* mRNA levels in the liver were calculated in comparison with *Gapdh* mRNA levels.

### Evaluation of adverse effects

Mice were intravenously injected with a 13-mer Toc-HDO (No. 1 or 18) at different doses. After 72 h, blood samples were collected for serum chemistry analyses and livers were collected for pathological studies; livers were stained with hematoxylin and eosin (Muto Pure Chemicals, Tokyo, Japan).

To examine whether the BBB was disrupted, mice were intravenously injected with 13-mer Toc-HDO (No. 18) at 50 mg/kg four times at 1-week intervals, and 72 h later were injected with 80 μL of 2% Evans blue solution. After 24 h, the brains were obtained and examined macroscopically. For pathological studies, brains were collected 72 h after the last injection of Toc-HDO and were stained with hematoxylin and eosin.

### Functional assay of the OAT3 protein in brain

The injectable solution of ^99m^Tc-ECD (Neurolite) was purchased from FUJIFILM RI Pharma (Tokyo, Japan). Nine volumes of saline were added to Neurolite, and 100 μl of the diluted solution (0.2 mBq of ^99m^Tc-ECD) was injected into mice via the tail vein. Because radioactivity associated with the brain showed first-order elimination following administration of ^99m^Tc-ECD by 30 min after the injection^16^, the brains were obtained 2 or 30 min after the injection and were homogenized. The radioactivity of the ^99m^Tc-ECD metabolite in the homogenates was measured with a gamma counter (COBRA Quantum 5003, PerkinElmer, MI). The values were corrected taking into consideration the decay during measurement, and normalized by the injected dose. The radioactivity efflux rate was calculated from the absolute values of radioactivity at each time point, assuming first-order elimination.

### Preparation of the microvascular fraction of the brain and western blot analysis

The microvascular fraction of the brain was prepared as reported earlier^9^. Briefly, mouse brains were homogenized in PBS and centrifuged for 5 min at 800 × *g* at 4 °C. The pellet was suspended in a 15% dextran solution and centrifuged for 10 min at 4500 × *g* at 4 °C. The pellet was resuspended in 5 mmol/l PBS, incubated for 10 min, and centrifuged for 3 min at 2000 × *g* at 4 °C.

The pellet was solubilized in homogenization buffer (10 mmol/l Tris-HCl (pH 7.4), 150 mmol/l NaCl, 1 mmol/l EDTA, 4% CHAPS, 1× Complete protease inhibitor cocktail (Roche Diagnostics)). Kidney samples from wild-type and *OAT3*-knockout mice^45^ were used as a positive and negative control, respectively. Samples (40 μl) were mixed with 10 μl of 5× Laemmli sample buffer (Bio-Rad, Hercules, CA), and then denatured at room temperature for 30 min. Proteins were separated by electrophoresis in a 5–20% polyacrylamide gel (ATTO Corporation, Tokyo, Japan) and transferred onto polyvinylidene difluoride membranes. Blots were probed with antiserum raised in rabbits against rat OAT3^46^ (1:1,000) and then incubated with anti-rabbit secondary antibody (1:2,000, 113-035-003, Jackson ImmunoResearch, Jennersville, PA) labeled with horseradish peroxidase, or probed with anti-β-actin antibody (1:2,000, 017-24573, Wako Pure Chemical Industries) labeled with horseradish peroxidase. Blots were visualized with SuperSignal West Femto Maximum Sensitivity Substrate (Thermo Fisher Scientific) and analyzed in a ChemiDoc System (Bio-Rad).

### Statistical analysis

All data represent means ± s.e.m. Student’s two-tailed *t*-test was used to determine the significance of differences between two groups in qRT-PCR assays, western blot analyses, and functional assays of the OAT3 protein.

### Data availability

The datasets generated during and/or analysed during the current study are available from the corresponding author on reasonable request.

## Electronic supplementary material

Supplementary Information
