# Supplementary material for: Modulation of blood-brain barrier function by a heteroduplex oligonucleotide in vivo
Source: Sci Rep. 2018 Mar 12;8:4377. doi: 10.1038/s41598-018-22577-2 (PMC5847588; doi:10.1038/s41598-018-22577-2)
Supplement: Supplementary file 1 — Supplementary Information [file 41598_2018_22577_MOESM1_ESM.doc]

**Supplementary Information**

**Modulation of blood-brain barrier function by a heteroduplex oligonucleotide *in vivo***

Hiroya Kuwahara, Jindong Song, Takahiro Shimoura, Kie Yoshida-Tanaka, Tadahaya Mizuno, Tatsuki Mochizuki, Satoshi Zeniya, Fuying Li, Kazutaka Nishina, Tetsuya Nagata, Shingo Ito, Hiroyuki Kusuhara, Takanori Yokota

**
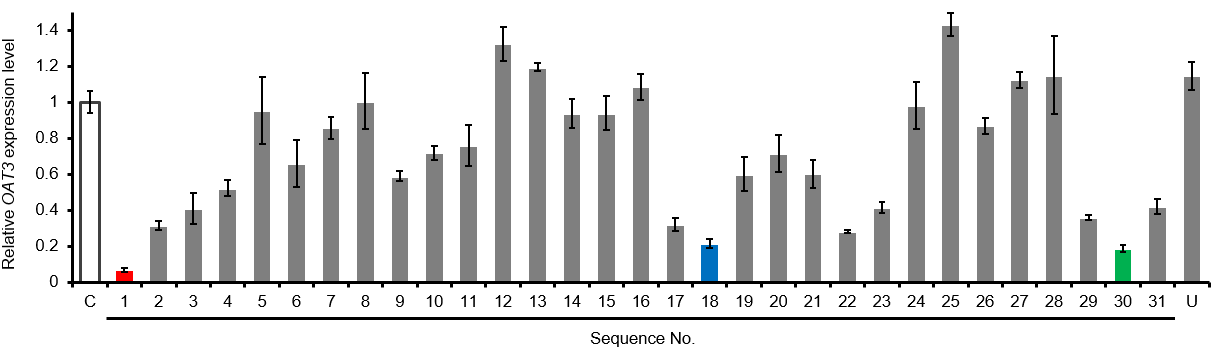
**

**Supplementary Fig. S1. Screening for ASO sequences that efficiently reduce mouse *OAT3* gene expression *in vitro.***

Luciferase activity was analyzed 24 h after transfection of Hepa1-6 cells with the *Renilla* luciferase-fused *OAT3* expression vector, firefly luciferase expression vector, and each ASO (10 nmol/l). C, cells transfected without ASO; U, cells transfected with an unrelated ASO. Data shown are relative to the values of the control group, and are expressed as mean values ± s.e.m. (*N* = 4).


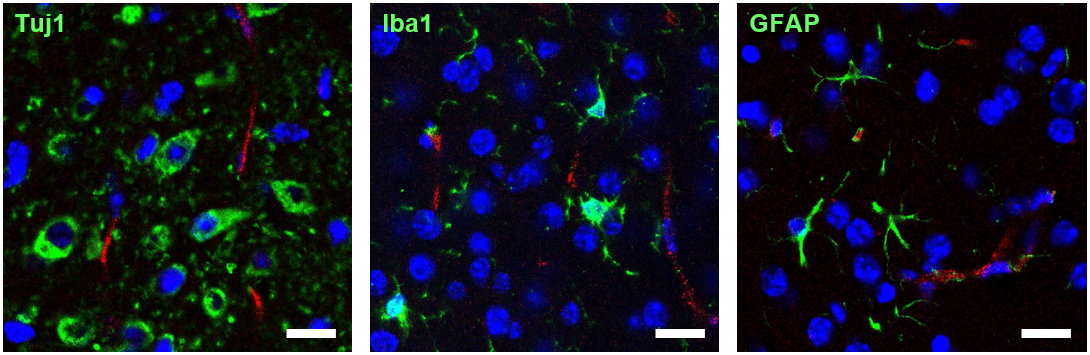


**Supplementary Fig. S2. Distribution of intravenously administered Toc-HDO in mouse brain.**

Confocal laser scanning images of cerebral sections prepared 1 h after an intravenous injection of Alexa Fluor 568–labeled Toc-HDO at doses corresponding to 16 mg/kg of ASO. Sections were stained with DAPI and were immunolabeled with antibody against Tuj1, Iba1, and glial fibrillary acidic protein (GFAP) to visualize neurons, microglia, and astrocytes, respectively. Red, Alexa Fluor 568. Scale bars: 20 µm.


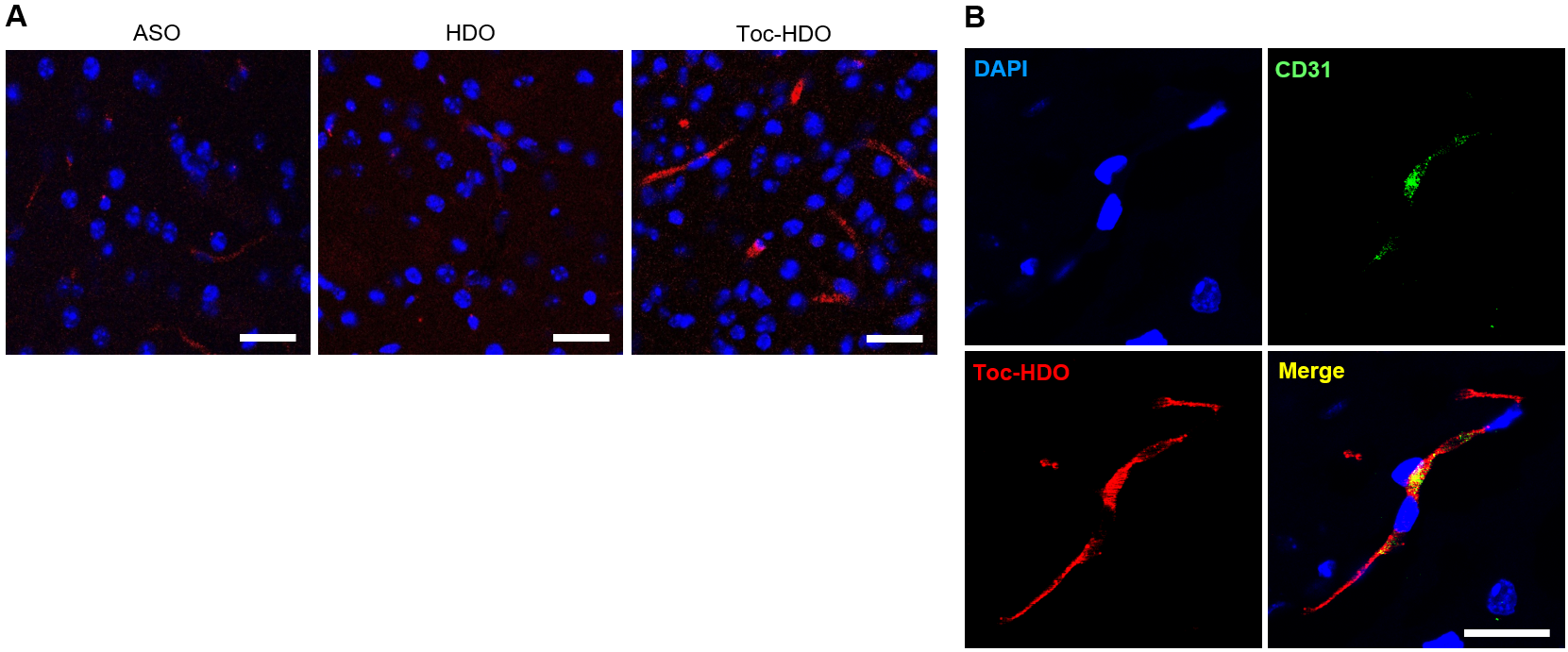


**Supplementary Fig. S3. Distribution of intravenously administered scramble**–**sequence oligonucleotides in mouse brain.**

Confocal laser scanning images of cerebral sections prepared 1 h after an intravenous injection of **(A)** Alexa Fluor 568–labeled scramble–sequence ASO, HDO, or **(A, B)** Toc-HDO at doses corresponding to 16 mg/kg of ASO. Sections were stained with DAPI and were immunolabeled with antibody against CD31 (**B** only). Red, Alexa Fluor 568. Scale bars: **(A)** 25 µm; **(B)** 10 µm.


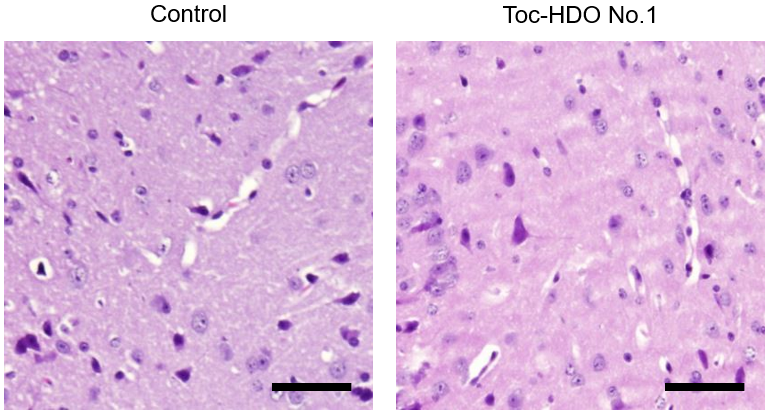


**Supplementary Fig. S4.** **Histology of the brain after an intravenous administration of Toc-HDO (No. 1).**

Hematoxylin and eosin staining of cerebral cortex sections. Mice were intravenously injected with 13-mer Toc-HDO (No. 1) at 16 mg/kg. Sections were prepared after 72 h. Scale bars = 100 µm.


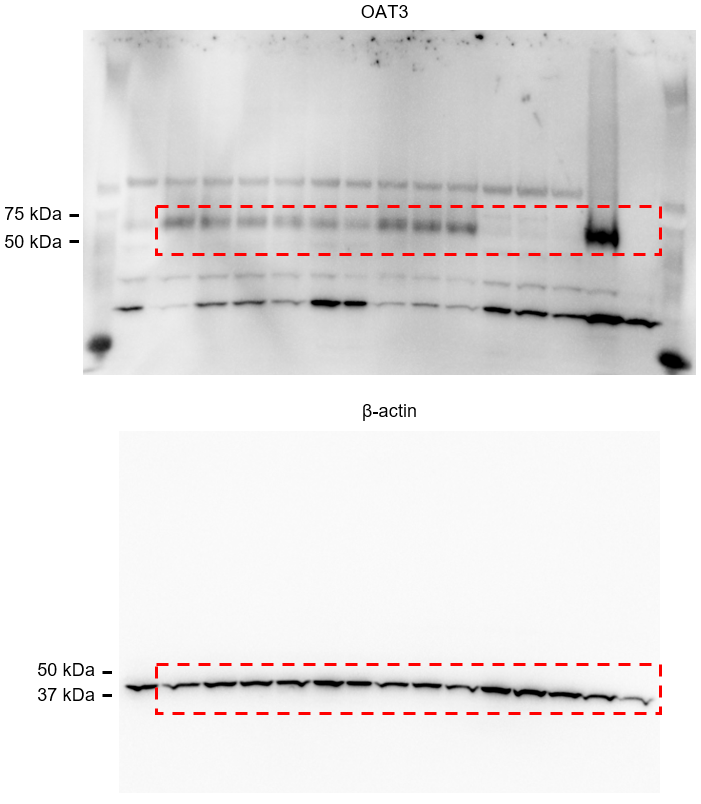


**Supplementary Fig. S5. Unprocessed scanned images of Figure 6B*.***

***
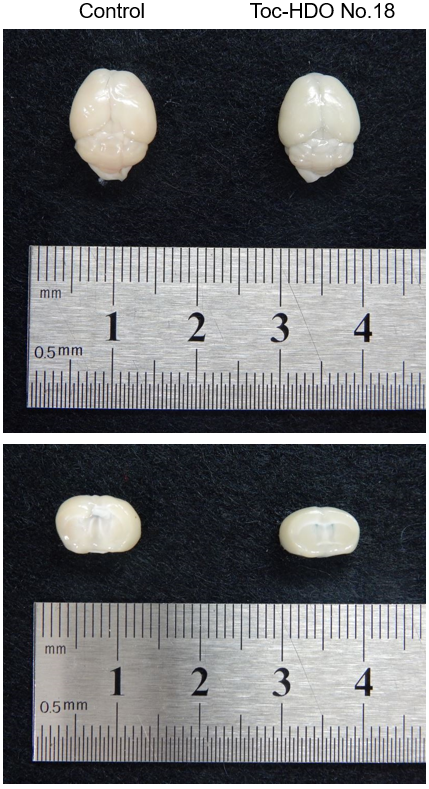
***

**Supplementary Fig. S6. Mouse brains after intravenous administration of Evans blue.**

Macrostructure of the brain (upper) and coronal section of cerebrum (lower). Mice were intravenously injected with 13-mer Toc-HDO (No. 18) at 50 mg/kg four times at 1-week intervals, followed 72 h later by intravenous injection of 80 μL of 2% Evans blue solution. Brains were collected after a further 24 h.


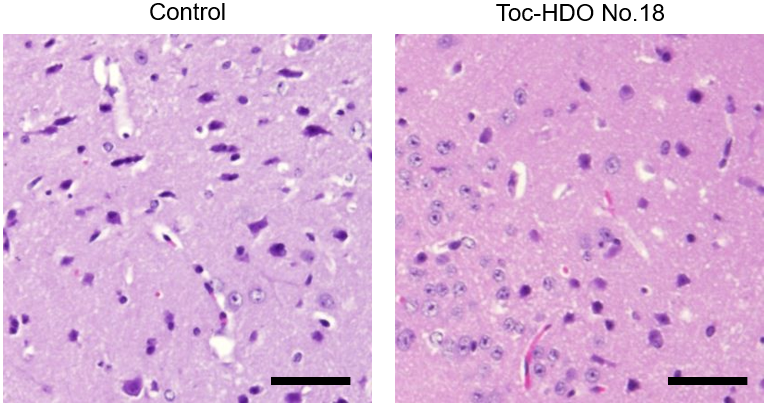


**Supplementary Fig. S7. Histology of the brain after repeated intravenous injections of Toc-HDO (No. 18).**

Hematoxylin and eosin staining of cerebral cortex sections. Mice were intravenously injected with 13-mer Toc-HDO (No. 18) at 50 mg/kg four times at 1-week intervals. Sections were prepared after a further 72 h. Scale bars = 100 µm.

***Supplementary Table S1. ASO sequences targeting mouse OAT3 mRNA for the experiments in vitro.***

| No. 1 | 5′-**G*****A****a***g***g***t***c***a***t***g****G*****C*****A**-3′ |
| --- | --- |
| No. 2 | 5′-**A*****T****g*c*t*t*c*c*a*a****C*****A*****C**-3′ |
| No. 3 | 5′-**T*****C****c*a*g*c*c*a*t*c****C*****A*****A**-3′ |
| No. 4 | 5′-**C*****T****g*a*g*a*g*c*a*t****G*****G*****A**-3′ |
| No. 5 | 5′-**A*****G****c*c*t*c*t*a*c*a****A*****C*****T**-3′ |
| No. 6 | 5′-**G*****T****g*g*t*g*a*a*g*g****T*****A*****A**-3′ |
| No. 7 | 5′-**C*****A****a*g*g*a*g*a*g*a****G*****T*****T**-3′ |
| No. 8 | 5′-**G*****T****g*a*a*a*t*c*t*g****G*****A*****G**-3′ |
| No. 9 | 5′-**G*****C****a*g*c*t*t*a*g*t****T*****C*****C**-3′ |
| No. 10 | 5′-**A*****G****a*a*c*c*a*g*c*t****T*****T*****G**-3′ |
| No. 11 | 5′-**A*****G****c*t*t*g*t*g*g*t****A*****G*****T**-3′ |
| No. 12 | 5′-**C*****A****a*a*g*g*a*a*g*a*t*t****A*****A**-3′ |
| No. 13 | 5′-**T*****C****a*a*a*g*g*a*a*g*a*t****T*****A**-3′ |
| No. 14 | 5′-**C*****G****a*g*t*g*a*c*c*t*g*a****G*****G**-3′ |
| No. 15 | 5′-**C*****T****g*g*a*t*t*c*a*g*t*t****G*****G**-3′ |
| No. 16 | 5′-**G*****A****t*g*a*a*c*c*a*a*a*a****C*****T**-3′ |
| No. 17 | 5′-**T*****G****t*c*t*g*a*c*a*g*t*t****C*****T**-3′ |
| No. 18 | 5′-**A*****A****a*c*c*t*g*t*c*t*g*a****C*****A**-3′ |
| No. 19 | 5′-**A*****T****g*g*a*c*t*c*t*g*g*t****A*****C**-3′ |
| No. 20 | 5′-**T*****A****t*g*g*a*c*t*c*t*g*g****T*****A**-3′ |
| No. 21 | 5′-**T*****A****t*g*t*a*g*a*t*g*t*t****G*****A**-3′ |
| No. 22 | 5′-**C*****G****g*g*a*a*t*g*t*c*a*a****C*****C**-3′ |
| No. 23 | 5′-**C*****C****t*t*c*c*c*a*a*a*t*a****C*****A**-3′ |
| No. 24 | 5′-**T*****G****c*c*a*g*g*g*a*a*t*c****T*****C**-3′ |
| No. 25 | 5′-**C*****A****g*g*g*a*a*t*c*t*c*a****A*****A**-3′ |
| No. 26 | 5′-**T*****G****g*t*g*a*t*a*g*a*a*a****G*****C**-3′ |
| No. 27 | 5′-**C*****A****t*g*g*t*g*a*t*a*g*a****A*****A**-3′ |
| No. 28 | 5′-**T*****T****t*a*a*g*t*t*g*t*t*t****A*****C**-3′ |
| No. 29 | 5′-**C*****T****t*c*c*c*g*t*t*t*g*g****G*****C**-3′ |
| No. 30 | 5′-**C*****T****g*g*g*t*g*t*c*a*t*t****G*****G**-3′ |
| No. 31 | 5′-**A*****T****t*a*g*g*g*a*t*g*a*a****G*****G**-3′ |
| Unrelated | 5′-**T*****G****t***c***c***a***g***a***t***a****T*****A*****C**-3′ |

Lowercase italic letters represent DNA, uppercase bold letters represent LNA (capital C denotes LNA methylcytosine) and asterisks represent phosphorothioate linkages.

***Supplementary Table S2. HDO sequences targeting mouse OAT3 mRNA for the experiments in vivo.***

| No. 1 | 13-mer | ASO | 5′-**G*****A****a***g***g***t***c***a***t***g****G*****C*****A**-3′ |
| --- | --- | --- | --- |
|  |  | cRNA | 5′-u*g*c*CAUGACCU*u*c-3′ |
|  | 14-mer | ASO | 5′-**A*****G*****A****a***g***g***t***c***a***t***g****G*****C*****A**-3′ |
|  |  | cRNA | 5′-u*g*c*CAUGACCU*u*c*u-3′ |
|  | 16-mer | ASO | 5′-**G*****A*****G****a***a***g***g***t***c***a***t***g***g****C*****A*****C**-3′ |
|  |  | cRNA | 5′-g*u*g*CCAUGACCUU*c*u*c-3′ |
| No. 18 | 13-mer | ASO | 5′-**A*****A****c*c*t*g*t*c*t*g****A*****C*****A**-3′ |
|  |  | cRNA | 5′-u*g*u*CAGACAGG*u*u-3′ |
|  | 14-mer | ASO | 5′-**A*****A*****A****c*c*t*g*t*c*t*g****A*****C*****A**-3′ |
|  |  | cRNA | 5′-u*g*u*CAGACAGG*u*u*u-3′ |
|  | 16-mer | ASO | 5′-**C*****A*****A****a***c*c*t*g*t*c*t*g*a****C*****A*****G**-3′ |
|  |  | cRNA | 5′-c*u*g*UCAGACAGGU*u*u*g-3′ |
| No. 30 | 13-mer | ASO | 5′-**T*****G****g*g*t*g*t*c*a*t****T*****G*****G**-3′ |
|  |  | cRNA | 5′-c*c*a*AUGACACC*c*a-3′ |
| Scramble | 13-mer | ASO | 5′-**A*****T****a***c***c***g***a***t***c***g****T*****C*****A**-3′ |
|  |  | cRNA | 5′-u*g*a*CGAUCGGU*a*u-3′ |

Lowercase italic letters represent DNA, uppercase bold letters represent LNA (capital C denotes LNA methylcytosine), uppercase letters represent RNA, lowercase letters represent 2′-*O*-methyl sugar modiﬁcation, and asterisks represent phosphorothioate linkages.

***Supplementary Table S3. Serum chemistry analysis after intravenous administration of Toc-HDO.***

|  | | T-Bil (mg/dl) | AST (U/l) | ALT (U/l) | ALP (U/l) | BUN (mg/dl) | Cre (mg/dl) |
| --- | --- | --- | --- | --- | --- | --- | --- |
| Control | | 0.063 ± 0.003 | 82 ± 14 | 54 ± 6 | 611 ± 8 | 20.6 ± 1.2 | 0.117 ± 0.003 |
| No. 1 | 2 mg/kg | 0.053 ± 0.003 | 115 ± 13 | 68 ± 10 | 612 ± 26 | 19.3 ± 1.4 | 0.127 ± 0.003 |
|  | 8 mg/kg | 0.063 ± 0.003 | 2340 ± 541 | 4057 ± 895 | 989 ± 134 | 20.9 ± 1.0 | 0.143 ± 0.003 |
|  | 16 mg/kg | 1.076 ± 0.359 | 16990 ± 4066 | 19330 ± 2935 | 754 ± 65 | 20.6 ± 1.2 | 0.160 ± 0.000 |
|  | 32 mg/kg | 2.010 | 27700 | 31400 | 1270 | 52.1 | 0.200 |
| No. 18 | 2 mg/kg | 0.100 ± 0.006 | 51 ± 2 | 30 ± 1 | 566 ± 15 | 31.0 ± 0.8 | 0.123 ± 0.003 |
|  | 8 mg/kg | 0.063 ± 0.009 | 57 ± 3 | 44 ± 4 | 552 ± 9 | 32.2 ± 0.1 | 0.127 ± 0.003 |
|  | 16 mg/kg | 0.070 ± 0.012 | 51 ± 7 | 28 ± 2 | 458 ± 58 | 34.9 ± 1.3 | 0.140 ± 0.006 |
|  | 32 mg/kg | 0.040 ± 0.006 | 121 ± 45 | 75 ± 1 | 586 ± 5 | 32.3 ± 1.9 | 0.153 ± 0.003 |
|  | 50 mg/kg | 0.073 ± 0.007 | 109 ± 12 | 85 ± 8 | 623 ± 25 | 28.9 ± 1.1 | 0.137 ± 0.009 |

T-Bil, total bilirubin; AST, aspartate aminotransferase; ALT, alanine aminotransferase; ALP, alkaline phosphatase; BUN, blood urea nitrogen; Cre, creatinine. Blood could be collected only from one of the three mice injected with 32 mg/kg of Toc-HDO (No. 1). Values represent mean ± s.e.m. (*N* = 3).
